# An Atypical Presentation of Henoch-Schönlein Purpura With Features of Acute Hemorrhagic Edema of Infancy and Koebnerization: A Case Report

**DOI:** 10.7759/cureus.79179

**Published:** 2025-02-17

**Authors:** Amira Basyouny, Ahmad Imam, Kamal A Abouzaid

**Affiliations:** 1 Department of Dermatology, Cairo University, Cairo, EGY; 2 Department of Anatomical Sciences, William Carey University College of Osteopathic Medicine, Hattiesburg, USA; 3 Department of Anatomical Sciences, Cairo University, Cairo, EGY

**Keywords:** acute hemorrhagic edema of infancy, atypical presentation, dermatology, henoch-schönlein purpura (iga vasculitis), koebnerization, pediatrics

## Abstract

Immunoglobulin A vasculitis, also known as Henoch-Schönlein purpura (HSP), is a type of vasculitis that is most commonly found in children. Palpable purpura is a key feature and is often associated with joint, abdominal, or renal involvement. This case report presents an atypical presentation of HSP in a four-year-old male, characterized by overlapping features of Acute Hemorrhagic Edema of Infancy (AHEI) and Koebnerization (isomorphic response), which complicates the diagnosis. The patient initially presented with redness around his mouth and nose, followed by hematuria and the development of palpable purpura on the lower extremities and buttocks. Vascular swellings were noted on the palms, feet, and face, and cockade purpura appeared on the upper extremities. The patient also exhibited episodic joint pain, genital edema, and Koebnerization on the lower back and buttocks. The diagnosis of this case was challenged by the presence of AHEI-like features and Koebnerization. The patient responded well to treatment with prednisone and colchicine. This atypical case presentation of HSP underscores the clinical overlap between HSP and AHEI, as well as the rare occurrence of Koebnerization in HSP. It emphasizes the importance of recognizing atypical presentations to ensure timely and accurate diagnosis. Further research is required to explore the frequency, pathophysiology, and clinical implications of such cases to enhance diagnostic accuracy and management in pediatric vasculitis.

## Introduction

Immunoglobulin A (IgA) vasculitis, also known as Henoch-Schönlein purpura (HSP), is a type of vasculitis predominantly affecting children [1]. This condition is characterized by non-thrombocytopenic, small-vessel vasculitis. It usually presents acutely, with an incidence rate of 3-27 cases per 100,000 children [2].

Palpable purpura is a key feature of this condition and is always present. In addition to joint, abdominal, or renal involvement [3], it may also affect other organs, such as the brain, lungs, heart, eyes, and testicles, though less frequently. HSP is a mild, self-limiting condition; it may resolve spontaneously in two weeks in one-third of cases, within two to four weeks in another third, and take more than four weeks in the remaining cases. The long-term outlook is associated with renal involvement [4].

Acute Hemorrhagic Edema of Infancy (AHEI), also known as postinfectious cockade purpura, is an acute cutaneous leukocytoclastic vasculitis first described in 1913 by Snow [5]. This condition typically affects children between 4 and 24 months of age and exhibits a triad of fever, substantial palpable purpuric skin lesions, and edema [6]. Its presentation is dramatic in appearance and has a sudden onset [7]. It starts with well-demarcated, annular, medallion-like purpuric plaques, almost entirely limited to the extremities and face, with relative sparing of the trunk. Additional features of AHEI include fever and painful edema of the distal extremities, ears, and eyelids [8].

The Koebner phenomenon, initially documented by German dermatologist Heinrich Koebner in 1876 [9], is an isomorphic response that describes the occurrence of a pre-existing skin condition in previously unaffected skin, triggered by internal or external injury [10]. The Koebner phenomenon is associated with psoriasis, lichen planus, and vitiligo, but, to our knowledge, has only been reported a handful of times to be associated with HSP [11].

This case study reports an atypical presentation of HSP featuring characteristics of AHEI and Koebnerization, highlighting the potential complexity of diagnosing this condition in pediatric patients.

## Case presentation

**Figure 1:**
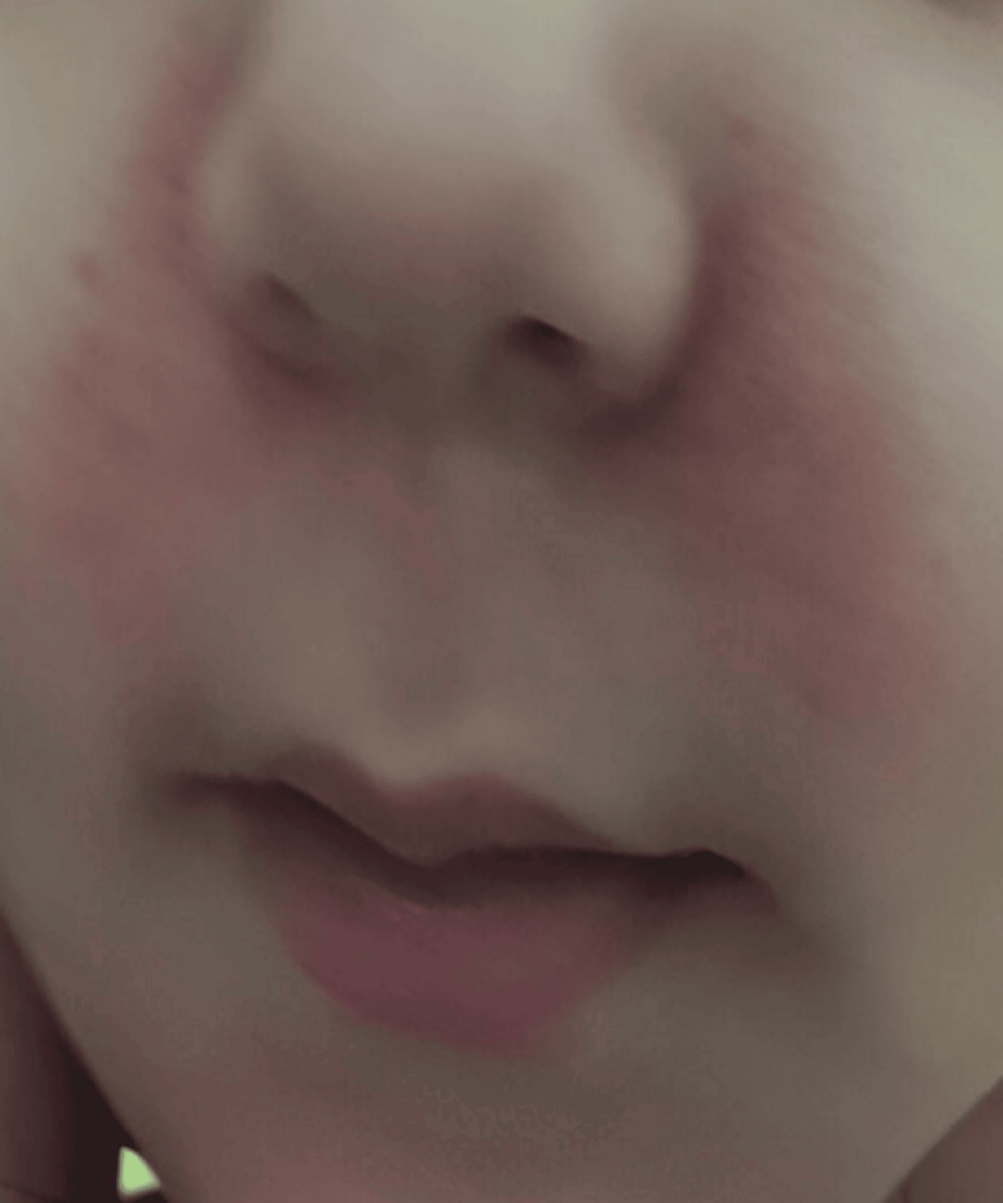
Erythema around the nose and mouth.

A four-year-old boy with no significant past medical history, presenting with redness around his mouth and nose (Figure 1) along with hematuria, presented to the pediatric clinic, where vitals were normal, and an abdominal ultrasound and coagulation profile testing were done and came back negative. The parents were advised for a follow-up visit.

**Figure 2:**
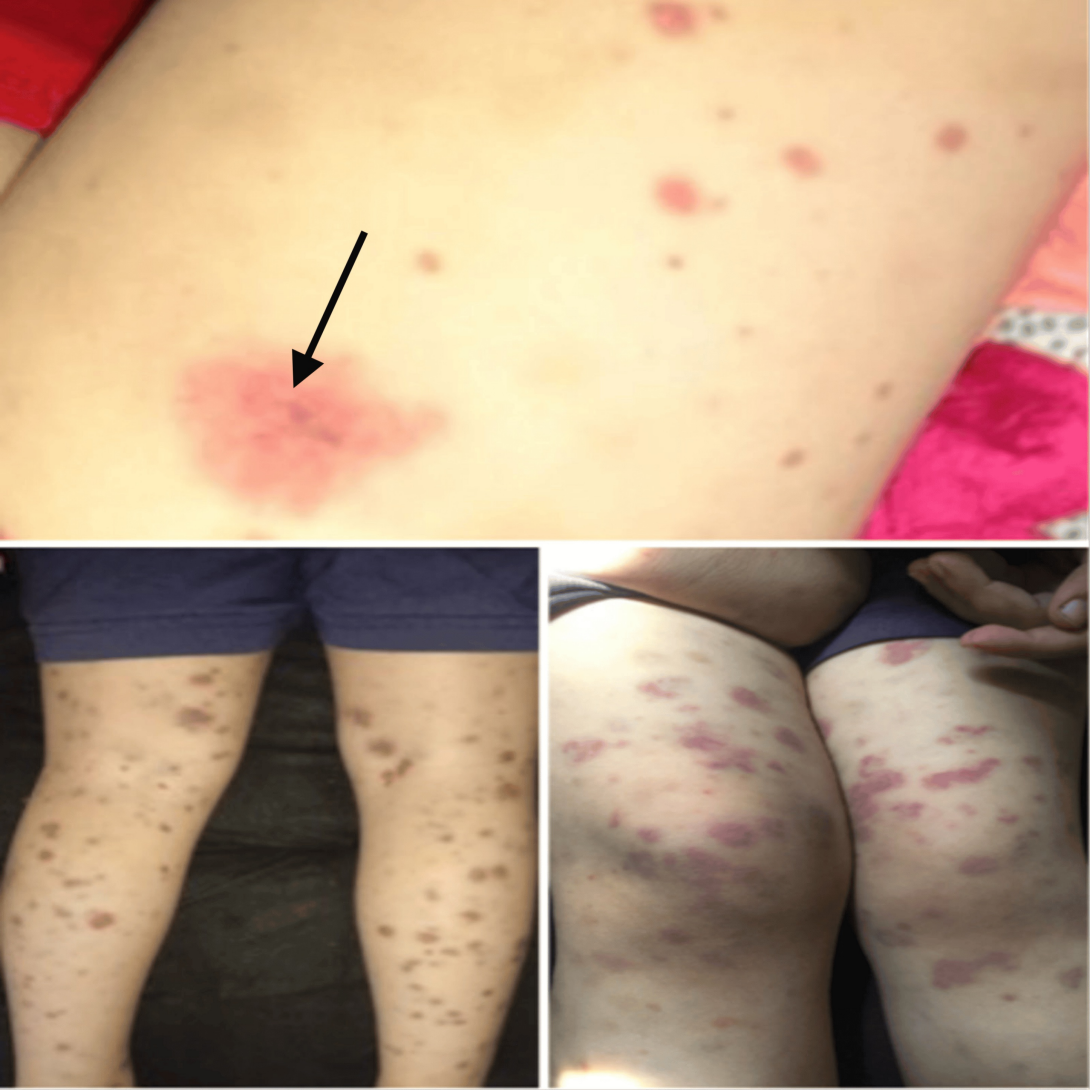
Palpable purpura of both lower limbs with signs of erosion and necrosis (black arrow).

**Figure 3:**
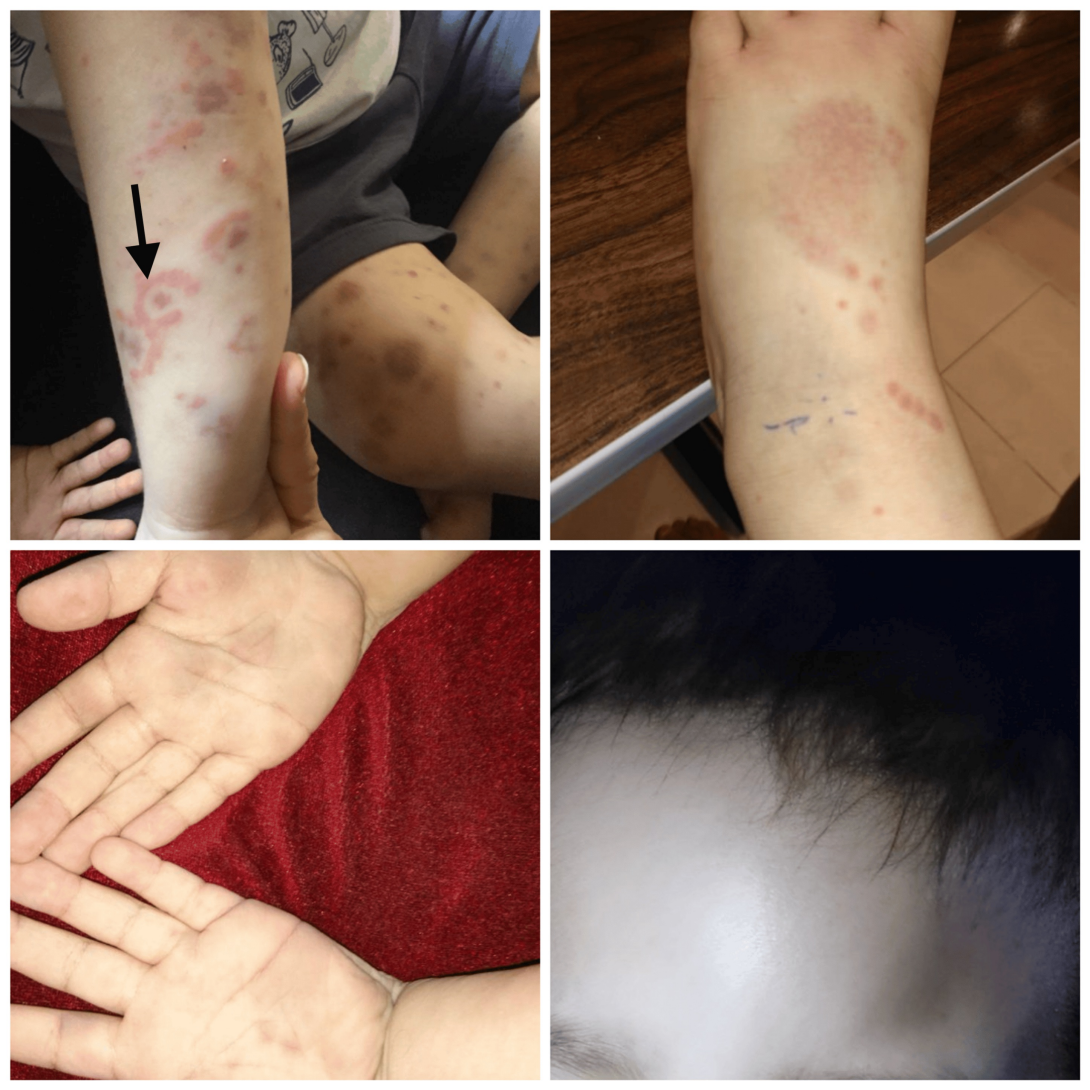
Swelling of the forehead, palms, and dorsum of the foot, accompanied by cockade purpura (large, round, and purplish skin lesion with an annular targetoid pattern) (black arrow) in the forearm of both upper limbs and dorsum of the foot.

Two days later, palpable purpura appeared in the lower extremities and buttocks (Figure 2). At the same time, the patient developed vascular swellings on his palms, feet, and face, accompanied by cockade purpura in the upper extremities (Figure 3). The vascular swellings led to suspecting Kawasaki disease, although there was no fever at the time. An echocardiogram was done for reassurance, and no abnormalities were detected. The patient was then referred to the dermatology clinic.

**Figure 4:**
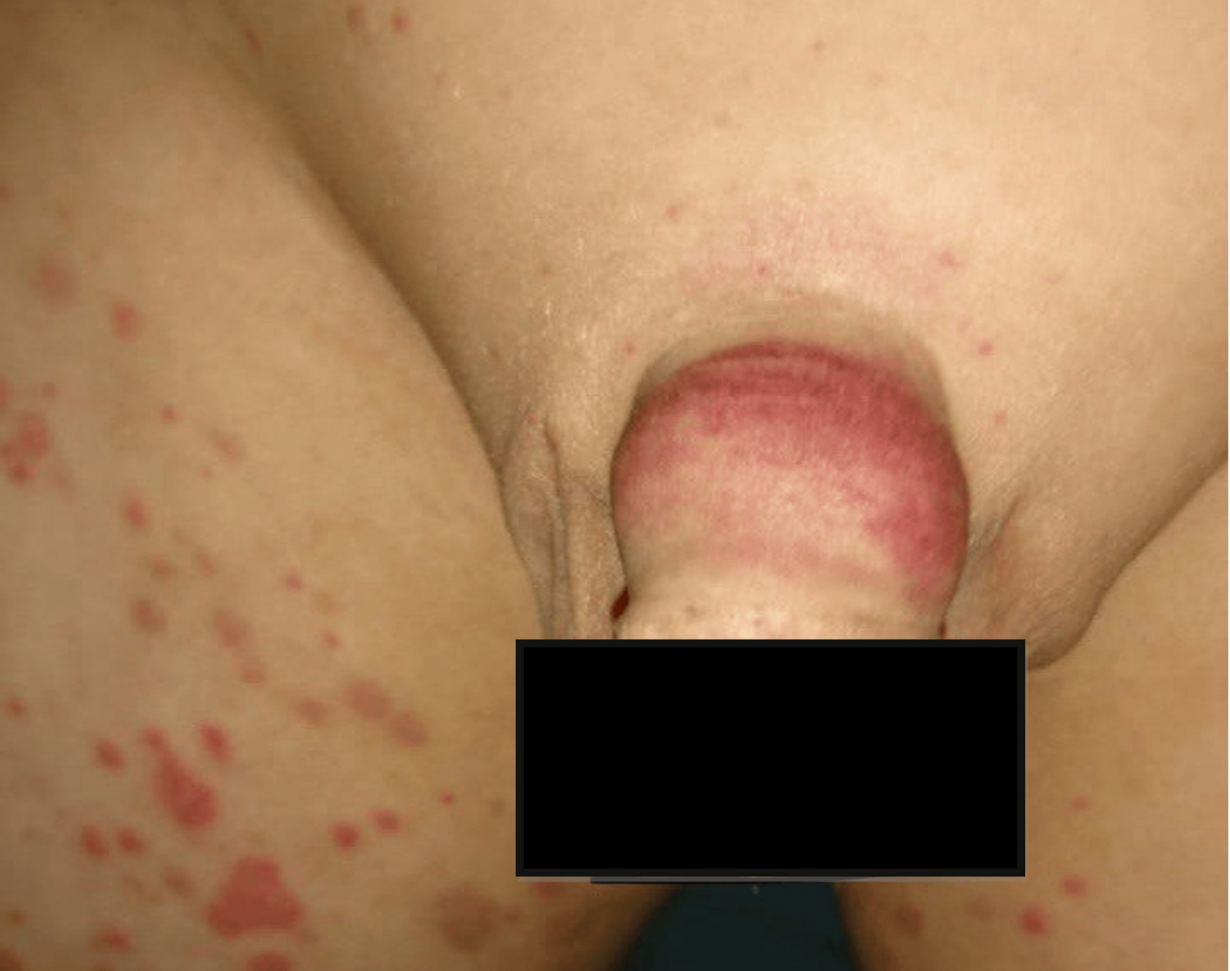
Redness and edema of the external genitalia.

Four days later, the patient came to the dermatology clinic complaining of joint pain and swelling, notably in the knee and hip, along with redness and edema of the genitalia (Figure 4).

**Figure 5:**
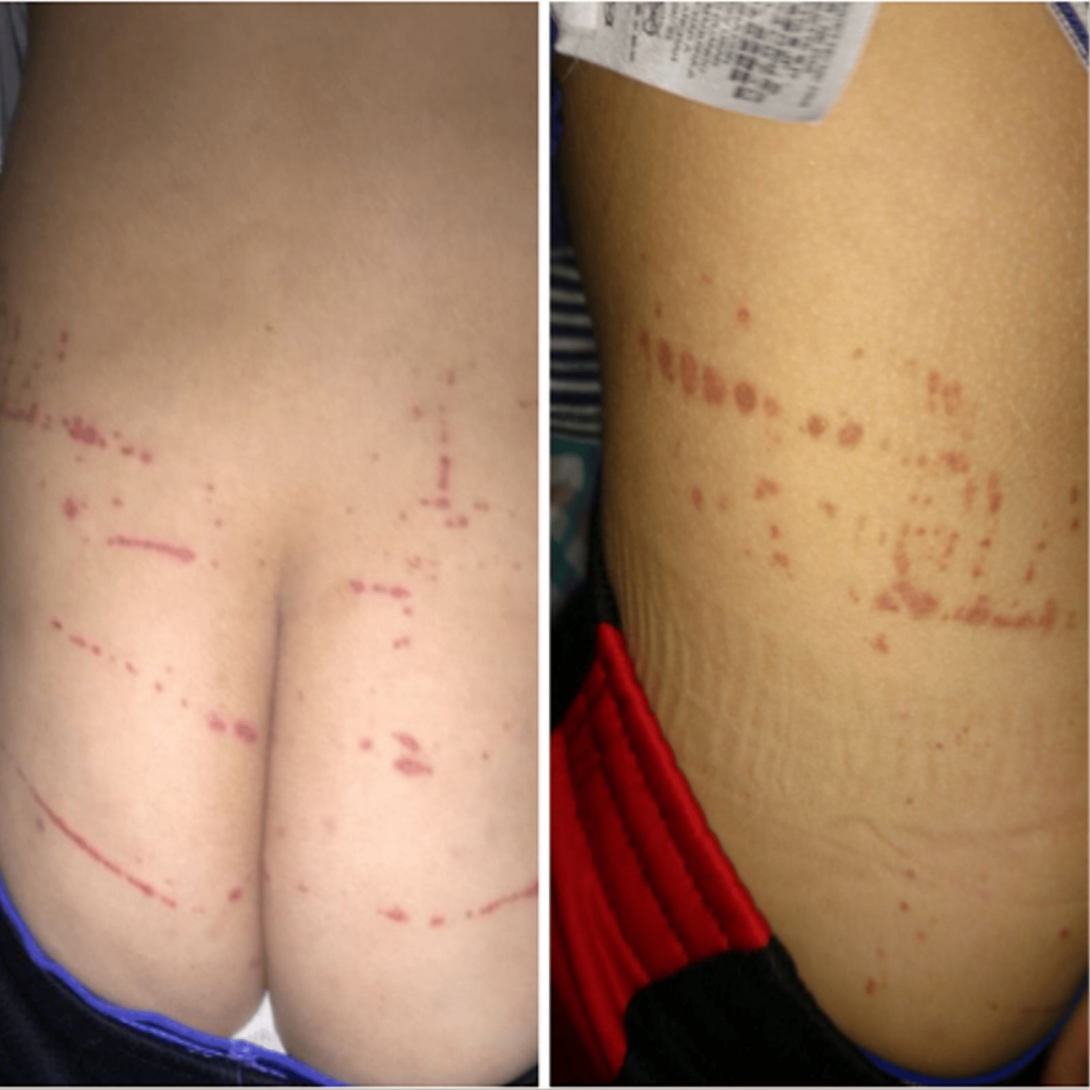
Koebnerization of the buttocks and lower back region caused by the compression of the elastic band of the clothes on the skin.

These symptoms occurred in episodic flare-ups. Additionally, the patient exhibited Koebnerization over the buttocks and lower back, at areas of scratching or somewhat tight clothes (Figure 5).

An atypical HSP was suspected due to palm, foot, and face vascular swellings, and purpura in the upper extremities, along with Koebnerization. Direct immunofluorescence and biopsy were consistent with the diagnosis of HSP. The skin biopsy report revealed, under the light microscope, that the upper dermis exhibited a mixture of hemorrhage and leukocyte infiltration. There was significant leukocytoclasia, with nuclear fragments from dying leukocytes mixed with intact ones, including many neutrophils. Three small vessels showed signs of mural fibrinoid necrosis. Immunofluorescence microscopy demonstrated granular staining of the vessel walls with an antibody specific to IgA.

Laboratory findings

Erythrocyte sedimentation rate (ESR) was mildly elevated (13 mm), and complement C3 and C4 were within normal limits. Complete blood count (CBC) showed moderate absolute lymphopenia (1600 uL) and mild absolute monocytosis (1400 uL) (hemoglobin 11.30 g/dL, platelet count 290,000 uL). The coagulation profile was normal. Urinalysis revealed mild hematuria; however, renal function tests were normal.

Treatment

The patient, on the fifth day since the beginning of symptoms, was treated with prednisone 1 mg/kg per day for one week, followed by a dose-tapering schedule for two weeks. Additionally, colchicine was prescribed at 0.5 mg, divided into two doses. After one week of treatment, the patient showed significant clinical improvement in the form of healing skin lesions, joint swelling and pain subsiding, and vascular swellings of the hand, forehead, and dorsum of the foot resolving completely.

## Discussion

HSP is a disease primarily affecting the skin, joints, abdomen, and kidneys [12]. The rash appears as palpable purpura, and it is present in all patients [13]. Joint pain is experienced by more than 80% of patients, typically affecting the knees and ankles, while abdominal pain, usually colicky, is experienced by over 50% of patients [14]. Renal involvement is present in about 50% of HSP patients and can manifest as proteinuria, microscopic hematuria, and red blood cell casts. While renal involvement in HSP usually resolves spontaneously, chronic renal disease can occur in 13% of the cases [15].

HSP most commonly affects children between the ages of four and six, with 90% of cases diagnosed before the age of 10. It is extremely uncommon in infants. In pediatric cases, there is a slightly higher incidence rate among males (male-to-female ratio of 1.5:1), and the disease becomes less common as age increases [1].

Our case presents an atypical presentation of HSP, characterized by the classic features of palpable purpura, joint pain, and hematuria, as well as more unusual manifestations, such as vascular swelling in the palm, feet, and face, accompanied by cockade purpura on the upper extremities. These unusual features suggest AHEI. AHEI was once considered an HSP variant, but evidence demonstrates that they are separate entities [16]. However, our patient had overlapping features of both conditions. Additionally, the presence of Koebnerization, a phenomenon rarely reported in HSP, further complicates the diagnosis. According to the literature, Koebnerization in HSP has been reported only nine times, with two instances occurring in the pediatric population [17].

The pathogenesis of IgA vasculitis remains poorly understood; nevertheless, IgA has a notable impact [18]. Immune complexes of IgA antibodies are created when the body encounters an infection or medication. These complexes then adhere to the small blood vessels, typically capillaries, of the skin, joints, kidneys, and gastrointestinal tract. As a result, there is an increase in the release of inflammatory substances, like prostaglandins. Furthermore, the complement system may become active as C3-receptor lymphocytes attach to immune complexes and are placed in the vessel walls, adding to the overly strong inflammatory reaction [14]. The skin shows palpable purpura and petechiae due to immune complexes deposited in the skin [18].

A key differential diagnosis for HSP is AHEI; however, patients with overlapping features of both have also been reported by Legrain et al. [19]. Both disorders have histologic features of leukocytoclastic vasculitis, but AHEI typically lacks the perivascular IgA deposition that is seen in 100% of HSP cases. While some AHEI cases have shown IgA deposition, it is not as prevalent as in HSP [20,21]. Both AHEI and HSP may present with perivascular deposition of IgM, fibrinogen, and C3. Age also plays a crucial role in distinguishing the two conditions, with AHEI typically affecting children aged 4 to 24 months (with male predominance), while HSP is more common in children aged 3 to 10 years [8]. This is consistent with our patient's age, as he was four years old at the time of the presentation. Another distinguishing feature is the extent of organ involvement. AHEI is almost always confined to the skin, whereas HSP usually shows renal, joint, and gastrointestinal tract involvement [8,20].

HSP is characterized by polymorphic skin lesions in the form of palpable purpura on the extensor surfaces of the lower extremities, with a tendency to spare the face. However, our case showed edema in the face and scalp, which does not correlate with both HSP and AHEI. Arunath et al. reported edema of the face and scalp in a case of HSP and explained the occurrence due to the inflammation of vessels, which leads to fluid leakage [22].

It has not been fully explained what exactly causes Koebnerization in HSP. There has been a report by Namazi, suggesting that the increase in tryptase levels following trauma could be responsible for the Koebner phenomenon observed in psoriasis [23]. This could explain the site of the Koebnerization in our patient. It was observed in the waistline area due to the pressure of the elastic band of his underwear. Additionally, it has been demonstrated by Horsmanheimo et al. that mast cell tryptase and chymase might act as regulators of neurogenic inflammation in psoriatic skin [24]. As a result, tryptase could potentially have a significant role in the development of the Koebner phenomenon [25]. While not extensively researched in vasculitis, recent studies have revealed that immuno-enzyme-histological staining of tryptase (a marker for mast cell activation) was present in cutaneous allergic vasculitis [26] or HSP nephritis [27]. This could help us understand the occurrence of Koebnerization with HSP in our case. 

## Conclusions

This report contributes to the limited literature on atypical HSP associated with Koebnerization and underscores the need for further research to explore the frequency and implications of atypical presentations. A better understanding of these atypical presentations will help reduce delays in diagnosis and improve management. Furthermore, this case also illustrates the clinical overlap between HSP and AHEI, suggesting the possibility of co-occurrence in some cases. Additional studies are essential to clarify the relationship between these two conditions and how they may present concurrently, ultimately enhancing the accuracy and timeliness of diagnoses.
